# INTERVAL (investigation of NICE technologies for enabling risk-variable-adjusted-length) dental recalls trial: a multicentre randomised controlled trial investigating the best dental recall interval for optimum, cost-effective maintenance of oral health in dentate adults attending dental primary care

**DOI:** 10.1186/s12903-018-0587-2

**Published:** 2018-08-07

**Authors:** Jan E. Clarkson, Nigel B. Pitts, Debbie Bonetti, Dwayne Boyers, Hazel Braid, Robert Elford, Patrick A. Fee, Ruth Floate, Beatriz Goulão, Gerry Humphris, Ian Needleman, John D. T. Norrie, Fiona Ord, Marjon van der Pol, Craig R. Ramsay, David N. J. Ricketts, Helen V. Worthington, Linda Young, INTERVAL Trial Collaboration, Tony Anderson, Trevor Burke, Philip Dolan, Gail Douglas, Ruth Freeman, Ronald Gorter, Richard Herbert, Penny Hodge, Dirk Mettes, Wendy McCombes, Margaret Ross, Debbie White

**Affiliations:** 1 University of Dundee School of Dentistry, Dundee, UK; 2 Dental Innovation and Translation Centre, King’s College London Dental Institute, London, UK; 3 Health Economics Research Unit, University of Aberdeen, Aberdeen, UK; 4 Patient Representative; Faculty of General Dental Practitioners, London, UK; 5 Health Services Research Unit, University of Aberdeen, Aberdeen, UK; 6 School of Medicine, University of St Andrews, St Andrews, UK; 7 International Centre for Evidence-Based Oral Health, Unit of Periodontology, UCL Eastman Dental Institute, London, UK; 8 University of Edinburgh, Edinburgh, UK; 9 Division of Dentistry, School of Medical Sciences, University of Manchester, Manchester, UK

**Keywords:** Dental recall, Oral health, RCT, Primary care

## Abstract

**Background:**

Traditionally, patients at low risk and high risk of developing dental disease have been encouraged to attend dental recall appointments at regular intervals of six months between appointments. The lack of evidence for the effect that different recall intervals between dental check-ups have on patient outcomes, provider workload and healthcare costs is causing considerable uncertainty for the profession and patients, despite the publication of the NICE Guideline on dental recall. The need for primary research has been highlighted in the Health Technology Assessment Group’s systematic review of routine dental check-ups, which found little evidence to support or refute the practice of encouraging 6-monthly dental check-ups in adults. The more recent Cochrane review on recall interval concluded there was insufficient evidence to draw any conclusions regarding the potential beneficial or harmful effects of altering the recall interval between dental check-ups. There is therefore an urgent need to assess the relative effectiveness and cost-benefit of different dental recall intervals in a robust, sufficiently powered randomised control trial (RCT) in primary dental care.

**Methods:**

This is a four year multi-centre, parallel-group, randomised controlled trial with blinded outcome assessment based in dental primary care in the UK. Practitioners will recruit 2372 dentate adult patients. Patient participants will be randomised to one of three groups: fixed-period six month recall, risk-based recall, or fixed-period twenty-four month recall. Outcome data will be assessed through clinical examination, patient questionnaires and NHS databases. The primary outcomes measure gingival inflammation/bleeding on probing and oral health-related quality of life.

**Discussion:**

INTERVAL will provide evidence for the most clinically-effective and cost-beneficial recall interval for maintaining optimum oral health in dentate adults attending general dental practice.

**Trial registration:**

ISRCTN95933794 (Date assigned 20/08/2008).

## Background

Traditionally, patients at low risk and high risk of developing dental disease have been encouraged to attend dental recall appointments at regular intervals of six months between appointments, with the logic of prevention and early detection of disease [1]. The lack of evidence for the effect that different recall intervals between dental check-ups have on patient outcomes, provider workload and healthcare costs is causing considerable uncertainty for the profession and patients, particularly following the General Dental Council guidance for team working responsibilities around recall intervals [2] and despite the publication of the NICE Guideline [3]. The need for primary research has been highlighted in the Health Technology Assessment Group’s systematic review of routine dental check-ups [4], which found little evidence to support or refute the practice of encouraging 6-monthly dental check-ups in adults. The more recent Cochrane review on recall interval found only one trial, which was assessed as having a high risk of bias, with 185 participants and concluded there was insufficient evidence to draw any conclusions regarding the potential beneficial or harmful effects of altering the recall interval between dental check-ups [5]. The limited evidence from recent observational studies also supports the need for research. Evaluation of the Canadian Non-Insured Health Benefits (NIHB) program found clients with more ‘regular’ check-ups received a standard pattern of service but incurred greater expenditure than those with longer gaps between recalls [6]. Recent evidence from the Dutch health system suggests an increase in General Dentists applying an individualised recall interval from 49% in 2000 to 61% in 2005 and that these dentists provide more frequent periodontal screening than those using a fixed recall interval [7].

Many Clinical Commissioning Groups (CCGs) in England are now seeking to secure adherence to the NICE recall interval guideline as part of their clinical governance responsibilities when commissioning dental primary care services. However, the lack of direct evidence behind differing recall strategies complicates the adoption process, while uncertainty remains within CCGs and among dentists as to how best to implement the guidance in practice.

There is therefore an urgent need to assess the relative effectiveness and value for money of different dental recall intervals in a robust, sufficiently powered randomised control trial (RCT) in primary dental care.

### Trial aim

The aim of this study is to compare the effectiveness and cost-benefit of dental check-ups at different recall intervals (fixed-period six month recall, risk-based recall or fixed-period 24 m recall) for maintaining optimum oral health in dentate adults attending general dental practice.

### Objectives

The primary objectives are to compare (1) gingival inflammation and (2) oral health-related quality of life (OHRQoL) of dentate adults receiving a dental check-up at different recall intervals over a four year period:

1. Fixed-period six month recall
2. Risk-based recall
3. Fixed-period 24 m recall

The secondary objectives are to compare the effect of the three recall strategies on periodontal probing depths, dental caries, calculus, preventive and interventive dental treatment, patient anxiety, patient satisfaction with care, oral health knowledge, attitudes and behaviours. The cost-effectiveness and cost-benefit of the three recall strategies will also be assessed as part of the economic evaluation.

## Methods

This is a UK multi-centre, parallel-group, randomised controlled trial with blinded outcome assessment. The trial is set in Primary Dental Care, reflecting the setting within which the vast majority of dentistry is delivered in the UK.

### Trial interventions

The trial interventions are three recall intervals – a fixed-period extended 24 m recall interval, a Risk-Variable-Adjusted-Length recall interval (Risk-based recall) based on the NICE Guideline and a fixed-period conventional six month recall interval.

#### Fixed-period recall intervals

Patient participants allocated to the conventional six month recall interval and extended 24 m recall interval groups will attend their dentist at the scheduled time intervals for a ‘routine’ dental check-up. The content of this check-up will remain as per current practice. A recognised definition of a ‘traditional’ NHS dental check-up is clinical examination, advice, charting including monitoring of periodontal status and report [4]. For participants allocated to the fixed-period recall groups dentists will provide routine care using their current system for examination, record keeping and providing advice.

#### Risk-based recall

**Fig. 1:**
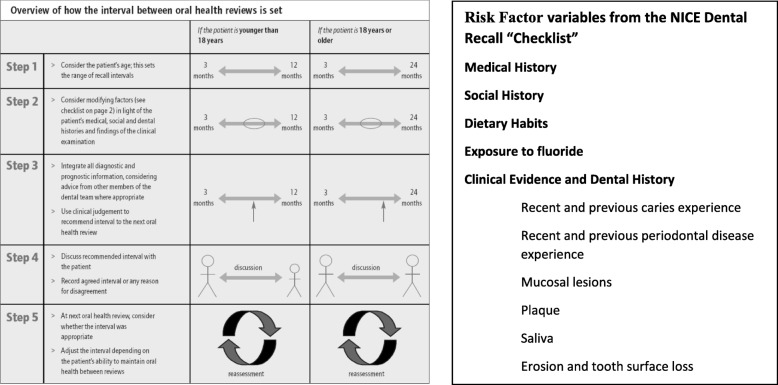
The process of recall interval selection

Participants allocated to the risk-based recall interval group will attend their dentist at time intervals determined by the evidence-based process outlined in the “2004 NICE guideline on Dental Recall” [2]. The essential steps of the procedure and the risk factors collected at recall examinations are outlined (from the Guideline) in the two figures below (Fig. 1).

The recommended stages in establishing the appropriate recall interval are:

1. **Establishing the Age Range** – establishing the individual patient’s age. In the case of this trial all patients will be adults of 18 years of age or more.
2. **Consideration of Risk Variables** – identification of the pertinent risk and protective factors present for each patient from the checklist and the Comprehensive Oral Health Examination, leading to the evaluation of the impact of these factors in the context of the patient’s past levels of oral health and current disease experience and then, consideration of a likely range of recall intervals.
3. **Integration and Prediction of Recall Need** - use of all of the information obtained by the dental team in order to predict the potential level of threat to maintaining oral health and controlling disease for this patient and, from this, to judge the most appropriate next recall interval.
4. **Discussion** – to explicitly discuss the recommended recall interval with the patient, explain the influencing factors in setting the recall and record the agreed interval (or any reason given by a patient in disagreement).
5. **Review** - at each check-up review (oral health review), the appropriateness of the just-concluded interval is reviewed by dentist and patient and the recall interval is reset according to the experience from the last period along with any change in the risk and protective variables identified at re-examination.

The frequency of recall interval appropriate for an individual patient will depend on the likelihood that specific diseases or conditions may develop or may progress beyond the control of secondary prevention. The guidance (built on extensive consensus methods and the limited evidence available) indicated that the recall interval range should vary from three to 24 m, according to risk. Training in assessment of relevant risk and protective factors to consider when allocating a recall interval will be provided by means of a specifically developed online training package based on the relevant NICE Guideline. Online training in allocation of risk-based recall will be carried out annually.

It is anticipated that by carrying out a comprehensive history and oral examination the dentist will be better informed to provide an accurate risk assessment and more appropriate preventive and interventive treatment recommendations leading to improvements in maintaining oral health, in oral health related quality of life and will result in less dental anxiety for the patient.

#### Recall strategy allocation

Prior to randomisation, all patient participants will be clinically examined by their dentist to determine suitability for randomisation to the 24 m recall arm. A detailed risk assessment is not part of this suitability examination and the decision made will be based on current practice, with routine examination criteria and record keeping.

Randomisation will be organised within two strata depending on the recruited participant’s suitability to enter the 24 m arm:

1) Twenty-four month recall versus risk-based recall versus six month recall;

2) Risk-based recall versus six month recall.

**Fig. 2:**
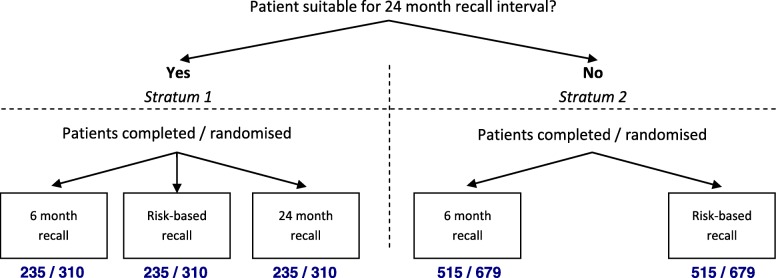
Study design

Randomisation to trial arms will be carried out using the automated central randomisation service at the Centre for Healthcare Randomised Trials (CHaRT) at the University of Aberdeen, with 24 h access by telephone. Allocation will take place after the suitability examination. Eligible participants will be randomised in equal proportions within each of the two strata according to a minimisation algorithm including dentist, age, filled teeth - FT (FT ≤ 8 or FT > 8 [8]) and absence of gingival bleeding on probing. There will be separate, identical algorithms for the two strata. The design is summarised in the graphic below (Fig. 2).

### Study recruitment and allocation

#### Identifying and recruiting dentists

We are aiming to recruit 50 general dental practitioners from across the UK, including dentists in England, Scotland, Wales and Northern Ireland, representing a cross-section of practitioners in terms of urban/rural area, community-level socio-demographics, and fluoridated or non-fluoridated communities. It is a requirement that all recruited dentists provide some NHS dental services for adults.

Dentists will be recruited to the trial through local research networks, advertising in the dental press, and trial team attendance at research network meetings. The Trial Office in Dundee (TOD) will send potential dentist participants an invitation letter, describing the study and interested dentists invited to a local information and recruitment session.

#### Identifying and recruiting patients

Recruitment of patient participants will be achieved through standard procedures and agreements for primary care research in the four nations. In Scotland co-ordinators from the Scottish Primary Care Research Network (SPCRN) will assist practice staff managing previously scheduled appointments, by including Patient Information Leaflets and an invitation to participate, in the appointment letters. In England, Wales and Northern Ireland appropriate regional Comprehensive Local Research Networks (CLRNs) will provide the identical service. The TOD will not have access to any data prior to the participant consenting to take part in the trial. At this stage, those who clearly express no interest in taking part will be sent an alternative check-up appointment to see their dentist. More than one dentist per practice can recruit participants into the trial. At the recruitment appointment the dentist will discuss the trial with the potential participants and answer any questions. Those who state they do not wish to take part will then receive their check-up as normal. Eligibility of those who express an interest in taking part will be confirmed against the trial inclusion and exclusion criteria. Those who are eligible will be consented to the trial by their dentist. The dentist will then examine the patient participant for suitability for randomisation to the 24 m arm.

### Inclusion criteria

Adult patients (≥ 18 years of age) who:

- Are dentate
- Have visited their dentist in the previous two years
- Receive their dental care as an NHS patient

### Exclusion criteria

- Patients who have a medical condition indicating increased risk of bleeding
- Immuno-compromised patients

### Outcome measures

#### Primary outcomes

##### Clinical

- Gingival inflammation/bleeding on probing at the gingival margin

##### Patient centred

- Oral health-related quality of life (OHRQoL)

#### Secondary outcomes

##### Clinical

- Dental caries
- Periodontal pocket depth
- Calculus
- Preventive and interventive care

##### Patient centred

- Dental anxiety
- Oral health related knowledge, attitudes, and behaviours
- Use of and reason for use of dental services
- Satisfaction with care

##### Service-providers

- Dentist attitude towards dental recall strategies

#### Economic outcomes

- NHS costs
- Patient incurred costs
- General population preferences, willingness to pay
- Net benefits (Benefits – costs)
- Generic Quality of Life, measured using the EQ-5D-3 L
- Quality adjusted life years (QALYs)
- Incremental cost per QALY gained

There are two primary outcomes: the primary clinical outcome is periodontal disease as measured by gingival bleeding, and the primary patient centred outcome is oral health-related quality of life as measured by the Oral Health Impact Profile-14 (OHIP-14). These are the outcomes that the study is powered at 80% to detect meaningful changes. Other measures are collected as secondary outcomes. Given the complex intervention and the unknown correlation between the two different dimensions of the study (patient and clinical), the study will not correct the *p*-value for multiplicity of tests.

### Data collection and processing

Participating dental practices will be expected to maintain a file of essential trial documentation which will be provided by the TOD. This will include documenting participant recall appointment attendance, dental treatment required, and duration of recall appointments.

### Collection of clinical outcome measures

Clinical outcomes will be measured at four years follow-up by trained outcome assessors who are blinded to allocation. Clinical outcome measures will be collected within four months either side of the four year anniversary of participant randomisation. Gingival inflammation as bleeding will be measured according to the Gingival Index of Loe [9] by running a UNC periodontal probe circumferentially around each tooth just within the gingival sulcus or pocket. After 30 s, bleeding will be recorded as being present or absent on the buccal and lingual surfaces. The colour-coded UNC periodontal probe will be used to measure periodontal pocket depth and then the presence of calculus. Clinical outcomes will be measured for all included teeth at six sites per tooth [mesiobuccal, midbuccal, distobuccal, mesiolingual, midlingual, and distolingual]. Third molar teeth will be excluded from clinical examination, except where a second molar tooth is absent and the third molar tooth has drifted mesially to occupy the position of the second molar. The sequence of scoring will be gingival inflammation, followed by periodontal pocket depths and calculus. The periodontal examination will take place first, then teeth brushed by the examiner and caries examination carried out.

Measurements of caries experience at the enamel and dentine threshold will be made using the validated International Caries Detection and Assessment System (ICDAS) [10–12] in the examination of clean and dry teeth aided by a ball-ended explorer that is used to remove any remaining plaque and debris and to check for surface contour, minor cavitation or sealants. Third molar teeth will be excluded from clinical examination, except where a second molar tooth is absent and the third molar tooth has drifted mesially to occupy the position of the second molar. All surfaces of all teeth excluding third molars will be examined and the caries status recorded.

Throughout the four year trial follow-up period recruited dentists will complete specially developed forms for each participant, documenting dental treatment provided during the trial period, including scheduled and re-scheduled check-up appointments. Details of preventive and interventive treatment provided during the trial period will also be accessed through the routinely collected data in all participating regions in the UK.

### Collection of patient centred outcome measures

Patient centred outcomes will be measured at baseline and annually by self-administered postal questionnaire. OHRQoL will be measured using the OHIP-14 [13]. A standardised measure of health status (EQ-5D-3 L) will be included to enable calculation of QALYs and conduct of the cost utility analysis. Patient dental anxiety status will be measured using recognised and validated psychological inventories [14, 15]. Annual patient questionnaires will also include questions relating to patient oral health related knowledge, attitudes and reported behaviours, satisfaction with dental care, and use of and reasons for use of dental services throughout the trial follow-up period.

### Collection of economic measures

The costs of all dental treatment provided by NHS dentists to patients, including the costs of planned check-ups are captured through the routinely collected data held by Information Services Division (ISD) in Scotland, NHS Information Centre, business services authority (BSA) in England and Health and Social Care Business Services Organisation (HSCBSO) in Northern Ireland. Time, travel and treatment costs associated with visits to the dentist will be collected through baseline questionnaires administered to trial participants on entry to the trial. Private dental care and use of other NHS services for dental issues are also collected by patient questionnaire.

Benefits to patients of the three intervention arms will be measured in two ways. Public preferences will be elicited regarding the value they place on different recall strategies using a discrete choice experiment (DCE) (see economic analysis). The DCE will be administered to a separate sample of the public obtained from electoral rolls over the course of the trial. Benefits are also measured in terms of QALYs (see above).

### Analysis plan

### Statistical analyses

The trial outcomes will be compared between the three trial arms using generalized linear models adjusting for the minimisation covariates and other covariates felt to be of prognostic importance. Statistical significance will be at the 2.5% level and corresponding confidence intervals will be derived. All participants will remain in their allocated group for analysis (intention to treat). Subgroup analyses will explore the possible effect modification of a number of factors including age, social class, residence in a fluoridated area, dentist characteristics (e.g. professional engagement, workplace stress) all using stricter levels of statistical significance (*p* < 0.01). All trial analyses will be according to a statistical analysis plan that will be agreed in advance by the Trial Steering Committee (TSC). The Data Monitoring and Ethics Committee (DMEC) will meet at 9, 24, 36 and 48 months to review progress and recommend any responses to divergences from planned trial design.

### Economic analyses

#### Estimation of costs

The costs will be estimated from both a NHS and a patient participant perspective. The NHS costs include the cost of the treatments provided by NHS dentists and primary care and secondary use related to dental issues. The participant incurred costs include time and travel costs for making return visits to the dentist and payments for care received.

The quantity, unit cost and average cost per participant will be reported. Regression analysis will be used to report differences in costs between randomised groups, adjusting for baseline participant characteristics (e.g. minimisation variables). Bootstrapping is used to obtain confidence intervals surrounding the average cost estimates.

#### Estimation of benefits

Two different approaches are used to estimate the benefits of recall strategies: Willingness-to-Pay and QALYs. The recall strategies may vary in terms of health related outcomes (e.g. caries) as well as non-health outcomes, such as level of reassurance. The benefits of the recall strategies are therefore valued in monetary terms - willingness to pay (WTP) value. A DCE will be used to provide a framework to weight different process and outcomes measures. Discrete Choice Experiments are increasingly used in the evaluation of health care interventions to produce overall benefit scores for treatments as well as examine the absolute and relative importance of different outcomes considered as important. This approach has been adopted as measures such as quality adjusted life years typically used in economic evaluations may not be sufficient to capture the strength of preferences for differences in the process and outcomes of care associated with each intervention. A DCE is used to estimate the WTP values for the different recall strategies. The DCE presents individuals with a series of choices between different scenarios which vary in terms of frequency of routine check-ups, experience of oral health problems, and cost. The DCE will be administered to a nationally representative sample of the UK general population.

The sample size required for the DCE reflects the need for the sample to be larger than the number of independent variables and provide an adequate sample for each pre-determined subgroup e.g. dental attendance (regular, non-regular), non-smoker or current smoker; socio-economic status (high, medium, low), (12 subgroups in total and 30–100 per subgroup). Allowing for individuals to be present in a number of groups, the questionnaire will be sent to 1000 individuals ([12 × 50]x[100/60]). Appropriately specified logistic regression analysis is used to model the preference as a function of the attributes. Marginal rates of substitution between the coefficients of the attributes and costs represent WTP.

Quality of Life is measured using the generic EQ-5D-3 L instrument. EQ-5D-3 L is a standardised instrument for use as a measure of health outcome. The EQ-5D-3 L scores are measured at baseline, and annually as part of the patient questionnaire. Quality Adjusted Life Years are estimated by estimating the area under the lines that link the utility scores obtained at the different time points. In case of any baseline differences in the EQ-5D-3 L scores, regression analysis is used where patient specific QALYs are modelled as a function of the baseline EQ-5D-3 L score and arm of the trial. The coefficient of arm of the trial represents the QALY differences adjusted for baseline differences.

#### Estimation of cost-effectiveness

The economic analysis will assess whether fixed-period 24-month or risk-variable-adjusted length dental recall represents an efficient use of NHS resources compared to the traditional fixed-period six-month recall. Cost-effectiveness will be measured in terms of the incremental cost per QALY gained and in terms of net benefits (cost-utility analysis and cost-benefit analysis).

The incremental cost per QALY is estimated by dividing the difference in mean total costs by the difference in QALYs between the arms of the Trial. This ratio can be compared to the conventional threshold of £30,000 per QALY. Bootstrapping is used to generate Cost-Effectiveness Acceptability Curves (CEAC). The CEAC shows the probability that the different recall strategies are cost-effective compared to usual care at various thresholds of cost-effectiveness. The threshold represents the decision-maker’s willingness to pay for an additional QALY. The CEAC represents the joint uncertainty surrounding the cost and QALY pairs.

The WTP values are mapped to recall strategy and trial outcomes and are directly compared with the costs to produce net benefits for each recall strategy. The recall strategy with the highest incremental net benefit compared to six-monthly recall is the most efficient option. A net benefit curve is produced which shows the probability of the net benefits being larger than different potential thresholds.

### Missing data

Two approaches will be used to deal with any missing data. The data will first be analysed using complete cases only. This may lead to biased and incorrect results if a relatively large proportion is missing. Multiple imputation using iterative chained equations is therefore also used to impute missing values. Data are imputed at each time point based on an iterative algorithm, imputing five datasets, adjusting for baseline characteristics. Data will be predicatively mean matched to fit within the range of the available data.

### Sensitivity analysis

Univariate sensitivity analysis is used to examine the impact of the discount rate, uncertainty surrounding the unit cost data, missing data, centre-specific variation.

### Sample size

An exploratory trial in a similar population reported 35% of gingival sites were bleeding on probing (standard deviation of 25%) [16]. The Cochrane review of periodontal instrumentation (PI) suggested that six monthly PI versus no PI reduces bleeding sites by 15% [17]. The recall interval would be expected to produce an effect lower than this given that the majority of participants in all arms will still receive PI at some time during follow up. Assuming either risk-based versus 24 m or six month versus 24 m could reduce/increase the proportion of sites bleeding by 7.5%, a study with 235 in each arm could detect such a difference with 90% power at 5% significance, and likewise detect a difference of 0.3 of the standard deviation of the OHIP-14 or any other global measure of OHRQoL. For the caries clinical outcome, assuming a standard deviation of 3.5 a study with 235 participants per arm could detect a shift in white spots lesions (from 3.3 to 4.2) at 80% power and 5% significance [18].

We can combine the two strata, without introducing bias, to estimate this comparison. We anticipate smaller effect sizes for the six month versus risk-based recall comparison than six monthly versus 24 monthly given that many of the participants in the risk-based group will be seen more frequently than 24 m. A study with 750 participants in each arm could detect a difference in bleeding scores of 4.5% at least 90% power and 5% significance level, and likewise detect a difference of 0.17 of the standard deviation of the OHIP-14. For the caries clinical outcome, assuming a standard deviation of 3.5 a study with 750 participants per arm could detect a 20% relative shift in white spot lesions from 3.3 to 3.9 at 90% power and 5% significance [18].

Although there is no reason to be concerned about contamination effects in this trial or clustering by dentist, the sample size has been conservatively estimated such that if contamination occurred with 15% of the control participants or the intra cluster correlation was 0.03, the study would still have 80% power to detect the hypothesised changes in the bleeding score. Our sample size calculations indicate we need to randomise 705 participants to stratum 1 (235 in each arm) and 1030 to stratum 2 (515 in each arm).

### Likely rate of loss to follow-up

In the power calculation we have assumed a loss to follow-up for dentists of 10% based on the observed rates of 12 and 9% in two recent large, multi-centre practice based RCTs [19]. For patient participants it is anticipated that loss to follow-up will be not more than 15% at 4 years. This estimate is based on a practice based trial conducted in North-West England where 79% of 4211 participants were retained for a period of 5 years [20]. In a recent trial conducted in a similar population 78% of participants returned a follow-up postal questionnaire. In this trial we anticipate a lower loss to follow-up because we will be using a more intensive reminder system and will explore the use of evidence based strategies to improve response.

### Ethical considerations

The trial will be coordinated by TOD in the University of Dundee and CHaRT in the University of Aberdeen – both centres with experience of multicentre trials, cognisant of the implications of research governance and other legal frameworks for the conduct of trials. Informed signed consent will be obtained from the trial participants who will be given sufficient time to accept or decline involvement and are free to withdraw from the study at any time. The design of the study ensures that adults for whom a 24-month recall interval may be detrimental are not put at risk of allocation to this group. It will be made clear to participants and dentists that participants may attend at any time if there is a need for a dental appointment between recall visits. No dental treatment including referral to specialist services will be withheld from participants as a result of taking part in the trial.

This is not classed as a trial of any investigational medicinal products or procedures, and so does not come under EU Clinical Trials Directive. We will continue to conduct the study to the standards required by the NHS Universities Research Governance Framework as well as all other applicable legal, ethical and regulatory requirements.

### Data protection and archiving

Participants will be reassured that all data which are collected during the course of the research will be kept strictly confidential. All participants’ details will be anonymised and stored on a database under the guidelines of the 1998 Data Protection Act. The relevant research documentation will be archived at the University of Dundee for at least five years after completion of the trial as required by the applicable regulatory requirement(s).

### Governance arrangements

Research Governance applies to everyone working in the TOD and CHaRT. All research will therefore be conducted within the appropriate legislative and regulatory environment and in accordance with Good Clinical Practice (GCP). All staff involved in the trial at the two centres will have undertaken appropriate GCP training. The two main groupings that contribute to the governance arrangements for this study are: an independent Trial Steering Committee (TSC); and an independent Data Monitoring Committee (DMC). The TSC includes an independent Chairperson and other independent members including a consumer representative/lay person. The TSC will also comprise a selection of the co-applicants including the joint Chief Investigators (Professor Pitts and Professor Clarkson), and the trial statistician (Professor Ramsay). There will only be two voting members drawn from any of the co-applicants. The TSC will meet annually throughout the course of the study. The Data Monitoring and Ethics Committee will continue to report any recommendations to the Chair of the TSC, meeting before the TSC. The Trial Management Committee (TMC) will include all co-investigators and act in an advisory capacity.

The University of Dundee has agreed to act as sponsor. As such, the TOD will undertake to communicate promptly and effectively with the sponsor to satisfy the sponsor’s obligations on the authorisations, financing and progress reporting of the trial are met.

### Arrangements for day to day management of the trial

The trial will be co-ordinated from the TOD and will provide day to day support for the dental practices. CHaRT will provide the database applications and IT programming for the TOD, host the randomisation system and take responsibility for all statistical aspects of the trial. The TOD will be responsible for transacting the randomisation, collecting all trial data, co-ordination of patient participant follow-up appointments, and data processing. The dental practice will be responsible for recruiting participants (including initiating the randomisation call). An Operations Management Committee, led by the Trial Manager, will meet weekly in the early stages at the TOD to ensure smooth running of the trial, trouble-shooting issues as they arise and ensuring consistency of action across the participating centres. CHaRT staff will join this group as required, weekly by teleconference and in person every 4–6 weeks. These face to face meetings will become less frequent as the trial progresses successfully, and increase again in frequency as the trial enters its closedown phase. The TMC will meet annually and be chaired by the Chief Investigators and include all the co-investigators and key members of the TOD and CHaRT. Their remit will be to oversee the progress of the trial in an advisory capacity.

### Publication

The results of the study will be reported first to study collaborators. A main report will be drafted by the project management group and circulated to all clinical co-ordinators for comment before a final version is considered for publication by the TSC.

### Dissemination

The results of this trial will be disseminated widely and actively through professional, primary care, public and scientific routes. Results will be communicated directly to all participating dental practices and an open workshop will be held with them discussing the next steps in getting the findings of the study to influence clinical practice. The trial results will be used to inform policy (through targeted feedback to all of the UK Health Departments and the British Association for the Study of Community Dentistry and its Consultants in Dental Public Health Group); practice (through specific communications to NICE, the British Dental Association and the Faculty of General Dental Practice (UK)); the public (through INVOLVE and patient organisations) as well as with dental education and training through a range of communications to postgraduate dental Deans and the undergraduate dental schools.

Given the current dearth of directly applicable evidence around this important research question it is anticipated that the impact of this trial will be felt at the International level as well as closer to home (specific presentations will be made to the International Association for Dental Research and its Evidence Based Dentistry Network as well as to organisations such as the European Association for Dental Public Health and related European specialty societies for research and practice).

## Discussion

The INTERVAL Dental Recalls Trial is an NIHR HTA funded trial being undertaken across the UK and will begin to address the lack of high quality evidence to aide dental practitioners, patients and policy makers in their decision making regarding allocation of dental recall intervals.

In order to ensure the results of this trial are widely applicable, the geographical areas that are included in the INTERVAL Trial have been selected to yield a cross-section of practices, operating in a range of different environments and circumstances (e.g. high, middle or low income communities, fluoridation status, rural and urban, method of remuneration of GDPs (capitation and fee for item of service or a banded payment system based on Units of Dental Activity (UDA)).

The study team is multidisciplinary and broad-based, which will ensure that whilst the trial design and conduct is of the highest standard, it remains practical and pragmatic at all times. We expect the INTERVAL Trial to provide evidence that will benefit the future dental care, improve outcomes of treatment and inform decision making by policy makers, clinicians and patients, within and out with the UK National Health Service.

## Abbreviations

BSA: Business services authority
CCG: Clinical commissioning groups
CEAC: Cost-effectiveness acceptability curves
CHaRT: Centre for healthcare randomised trials
CLRN: Comprehensive local research network
DCE: Discrete choice experiment
DMEC: Data monitoring and ethics committee
FT: Filled teeth
GCP: Good clinical practice
HSCBSO: Health and social care business services organisation
ICDAS: International caries detection and assessment system
ISD: Information services division
NHS: National health service
NICE: National institute for health and care excellence
NIHB: Non-insured health benefits
NIHR: National institute for health research
OHIP-14: Oral health impact profile-14
OHRQoL: Oral health-related quality of life
PI: Periodontal instrumentation
QALYs: Quality adjusted life years
RCT: Randomised control trial
SPCRN: Scottish primary care research network
TMC: Trial management committee
TOD: Trial Office Dundee
TSC: Trial steering committee
WTP: Willingness to pay
